# Preharvest Transmission Routes of Fresh Produce Associated Bacterial Pathogens with Outbreak Potentials: A Review

**DOI:** 10.3390/ijerph16224407

**Published:** 2019-11-11

**Authors:** Chidozie Declan Iwu, Anthony Ifeanyi Okoh

**Affiliations:** 1 SAMRC Microbial Water Quality Monitoring Centre, University of Fort Hare, Alice 5700, South Africa; aokoh@ufh.ac.za; 2 Applied and Environmental Microbiology Research Group, Department of Biochemistry and Microbiology, University of Fort Hare, Alice 5700, South Africa

**Keywords:** preharvest, irrigation water, agricultural soil, food safety, antibiotic resistance, environmental health, public health

## Abstract

Disease outbreaks caused by the ingestion of contaminated vegetables and fruits pose a significant problem to human health. The sources of contamination of these food products at the preharvest level of agricultural production, most importantly, agricultural soil and irrigation water, serve as potential reservoirs of some clinically significant foodborne pathogenic bacteria. These clinically important bacteria include: *Klebsiella* spp., *Salmonella* spp., *Citrobacter* spp., *Shigella* spp., *Enterobacter* spp., *Listeria monocytogenes* and pathogenic *E. coli* (and *E. coli* O157:H7) all of which have the potential to cause disease outbreaks. Most of these pathogens acquire antimicrobial resistance (AR) determinants due to AR selective pressure within the agroecosystem and become resistant against most available treatment options, further aggravating risks to human and environmental health, and food safety. This review critically outlines the following issues with regards to fresh produce; the global burden of fresh produce-related foodborne diseases, contamination between the continuum of farm to table, preharvest transmission routes, AR profiles, and possible interventions to minimize the preharvest contamination of fresh produce. This review reveals that the primary production niches of the agro-ecosystem play a significant role in the transmission of fresh produce associated pathogens as well as their resistant variants, thus detrimental to food safety and public health.

## 1. Introduction

Foodborne pathogens have been identified as significant causes of morbidities and mortalities, particularly in developing countries, and a huge sum of funds are spent on social and medical expenses [1]. Going by the annual estimates of the World Health Organization (WHO), 30% of the people in developed nations suffer from foodborne diseases, and up to 2 million deaths are recorded in developing countries [2]. The issue of food safety is progressively becoming a challenge to public health in many countries, and biological contaminants, particularly bacteria constitute the leading cause of foodborne illnesses [1]. 

Currently, epidemiological studies have linked a significant portion of foodborne illnesses to the ingestion of produce contaminated with foodborne pathogenic bacteria [3]. Fresh produce forms an essential component of a healthy diet which provides antioxidants, minerals, vitamins and other compounds that promote wellbeing [4]. However, foodborne illnesses linked to field-grown produce particularly leafy greens and vegetables have increased proportionally, resulting in substantial burden to public health and multiple disease outbreaks globally [5,6]. Some studies have proposed that this increase might be as a result of (i) personal consumption deviations, (ii) amplified livestock production near fresh produce production areas, (iii) rapid global availability of fresh produce, with some traced back to areas with unknown hygienic conditions, and (iv) increased numbers of immunosuppressed consumers [7,8]. The fact that fresh produce is usually eaten raw or slightly processed, further explains why they are important vehicles for the spread of pathogenic bacteria. These bacteria may have been initially linked to foods of human and animal source or linked to the farm environment [9].

Usually, fresh produce is grown on open fields where they are constantly exposed to preharvest microbial contamination through contaminated irrigation water, agricultural soil, raw or improperly composted manure and/or faeces deposited by intruding domestic or wild animals [10], which traverses the farm to table continuum. Fresh produce can also be contaminated through harvesting equipment, processing plants, field workers, and trading processes along the farm to table continuum at the postharvest stage [4]. Identifying the contamination sources at the preharvest stage is vital since the reduction or eradication of microbial contamination that happens before harvest is tough to attain during or after the postharvest stage [11]. 

Irrigation water and agricultural soils are the primary reservoirs and transmission routes of human pathogens at the preharvest stage because of the ability of these pathogens to survive for long within these two agrarian niches [4]. Irrigation water is significantly considered as a vital transmission route of pathogenic microbes to farm produce, because enteric pathogens from the soil, faecal materials, sewage overflow etc. are introduced continuously into the watercourse from where water for irrigation is usually extracted [12]. Water used for irrigation purposes is naturally sourced from surface waters, harvested rainwater, desalinated seawater, shallow groundwater and deep aquifers [13]. Due to some factors including economic, political and climate challenges, most farm owners have reverted to the use of raw or inadequately treated wastewater for the irrigation of their farm produce [14]. An alternative source of irrigation water is municipal water. Though it is believed to have a high microbiological standard because of the treatment processes it undergoes, its usage for irrigation purposes is discouraged in many countries because it is expensive and not generally available [15]. Agricultural soil also serves as a natural reservoir of enteric pathogens. This is exacerbated when bio-solids, sludge, manure and animal excrement are discharged onto soil surfaces during waste disposal or soil amendment [13]. Soil that contains manure of animal origin has higher chances of being a reservoir of enteric pathogens due to their propensity to subsist in soils for months or even years [8]. 

Although parasites and viruses are involved in fresh produce associated disease outbreaks, bacterial pathogens represent the major microbial hazard implicated in fresh produce linked foodborne illnesses [9]. Bacteria such as pathogenic *E. coli* (and *E. coli* O157:H7), *Klebsiella* spp., *Salmonella* spp., *Enterobacter* spp., *Citrobacter* spp., *Shigella* spp. and *Listeria monocytogenes* are more implicated [3,16,17]. In humans, these group of foodborne bacteria produces clinical syndromes that range from fever, mild diarrhea, headaches, vomiting, muscle cramps and abdominal pain to more complex problems like haemorrhagic colitis (*E. coli* O157:H7), enterotoxin poisoning (*E. coli* O157:H7, pathogenic *E. coli, Shigella* spp.), haemolytic uremic syndrome (HUS) (*E. coli* O157:H7), septicaemia (*Salmonella* spp.), dysentery (pathogenic *E. coli*, *Shigella* spp.), miscarriage in pregnant women (*Listeria monocytogenes*), autoimmune complications and meningitis (*Enterobacter* spp.) with the “at-risk” group including the infants, prenatal women, immunosuppressed and elderly being more affected [3].

Unfortunately, the reduction of infections caused by this group of pathogens is compromised by the constant evolution of antibiotic resistance. Some of these bacteria even resist the effects of extended-spectrum beta-lactams such as penicillin, cephalosporins and carbapenems, which are considered as antibiotics of last resort. Antibiotic resistance of this nature poses a greater risk to global health. Understanding the transmission routes of these pathogens and their fate at the preharvest level is imperative for the planning and execution of interventions aimed at reducing or even eliminating the contamination of fresh produce by bacterial pathogens. Although they vary in prevalence and may pose higher risks in some regions than others. To that effect, we did a thorough literature review on the global burden of fresh produce-related foodborne diseases, fresh produce contamination between the continuum of farm to table, preharvest transmission routes of fresh produce associated pathogens, selected pathogenic bacteria and their antibiotic resistance properties, and possible interventions to minimize the preharvest contamination of fresh produce. This review collates the current scientific information on the ecological pattern of foodborne pathogens at the preharvest stage, thus providing possible means of reducing or eliminating their transition and colonization of fresh produce. It also provides new knowledge on antimicrobial resistance trends in these pathogens which will provide information on the set of antimicrobials still useful for the alleviation of infections instigated by the pathogens.

## 2. Global Burden of Fresh Produce-Related Foodborne Diseases

Fresh produce including fruits and vegetables are the chief source of “micronutrients” for example minerals, polyphenolics, carotenoids, glucosinolates, vitamins and “macronutrients” such as carbohydrates and fibre [18]. Based on this, “The 2015–2020 Dietary Guidelines for Americans, published jointly by the U.S. Department of Agriculture” has encouraged both male and female consumers in the U.S. to increase their fresh produce consumption by over 100% which is about 9 servings (approximately 4.5 cups) for a 2000 calorie diet per day [19]. In European countries, for instance, Germany, people are encouraged to consume at least 650 g (400 g vegetables, 250 g fruits) of fresh produce per day [20]. As a result, fresh produce has gained popularity globally, and its market has increased explosively due to its demand [21]. The outbreak of illnesses linked to fresh produce has also increased globally [16], especially with leafy greens including cabbage, lettuce, spinach, and fresh herbs such as parsley and basil which are potential sources of infection-causing bacteria [22]. Other fresh produce considered to be at risk of contamination includes green onions, berries, melons, tomatoes and sprouted seeds [8,23].

Recently, agricultural produce represents a prominent source of foodborne disease outbreaks exceeding other microbial vehicles such as milk, seafood and meat [24]. In the United States of America, a large number of multistate fresh produce associated outbreaks have happened including the 2006 outbreak of *E. coli* O157:H7 connected to the ingestion of packaged spinach which caused three deaths and almost 200 food poisoning cases [25,26] as well as several other salmonellosis outbreaks caused by contaminated fresh tomatoes which has occurred since past ten years [27]. In total, 16 among 68 multistate foodborne disease outbreaks in the U.S. between 2006 and 2014 were fresh produce-related [28]. Between 1996 and 2010, about 23% of all foodborne outbreaks in the U.S. were also linked to fresh produce [4]. In Europe, 10% of foodborne outbreaks between 2007 and 2011 were linked to fresh produce causing about 35% hospitalizations and 46% mortalities [29]. The outbreak of salmonellosis, with 63 established cases of food poisoning in several countries in Europe between late 2011, and early 2012 were linked to watermelon from Brazil [30]. In Australia, about 4% of all associated foodborne outbreaks between 2001 to 2005 were linked to fresh produce [31]. In the UK, an outbreak of infection caused by *Salmonella* spp. linked to contaminated basil from Israel affected a minimum of 51 people from Scotland, England, U.S., Denmark, Wales, and the Netherlands as uncovered from the microbiological investigation of fresh herbs retailed in the UK in 2007 [32,33]. In Canada, over 1360 cases of foodborne illnesses resulting from 15 outbreaks between 1991 and 2000 were linked to fresh produce [34]. Most of the disease outbreaks associated with fresh produce are mostly reported in developed countries such as Australia, USA, UK and Canada, unlike the developing and underdeveloped countries such as African and Asian countries where there are no efficient surveillance techniques to carry out disease surveillance [35]. This is detrimental to the prevention and control of fresh produce associated disease outbreaks within these regions. Table 1 summarizes some foodborne disease outbreaks linked to fresh produce.

## 3. Fresh Produce Contamination between the Continuum of Farm to Table

The concept of “farm to table continuum” encompasses the stages involved in food production throughout the food chain [82], starting with the growing, processing, distribution and then to the consumption of food products. Usually, the production chain of fresh produce is multifaceted and comprises different necessary steps where microbial safety can be compromised, leading to fresh produce contamination by pathogenic microorganisms originating from either environmental, animal or human sources throughout the farm to table continuum [21,83]. Good Agricultural Practices (GAP) set by the United States Department of Agriculture (USDA) was designed to reduce the risks of fresh produce contamination by recommending best practices in areas such as irrigation water quality, manure management, wildlife management, worker health and hygiene and post-harvesting handling [84]. It is possible to reduce the risks through compliance with the GAP and proper risk assessment. However, due to the exposed nature of fresh produce cultivation chain, it is generally agreed and demonstrated that it is not possible to achieve a zero risk. Hence, fresh produce is continually exposed to microbial contaminants which are eventually passed to final consumers causing public health burden [85]. Pathways through which foodborne pathogens get into the fresh produce could either be before or after harvest [86]. Sources of contamination before harvesting include irrigation water, agricultural soil, reconstituted fungicides and insecticides, human and animal faeces, insects, dust, human handling and defectively composted manure. After harvesting, contamination sources include equipment used to harvest, transportation vessels, insect, dust, processing equipment, water used for washing of harvested produce, ice, vehicles as well as human handling [8]. As a result of certain factors like internalization of pathogens within the tissues of plants, plant surface hydrophobicity and formation of bacterial biofilm on plant surfaces [87], it is challenging to control or destroy fresh produce contaminants at both preharvest and postharvest stage, hence identification of routes of contamination on the farm and after harvest becomes very crucial to the control of fresh produce disease outbreak.

## 4. Preharvest Transmission Routes of Fresh Produce Associated Foodborne Pathogens

**Figure 1:**
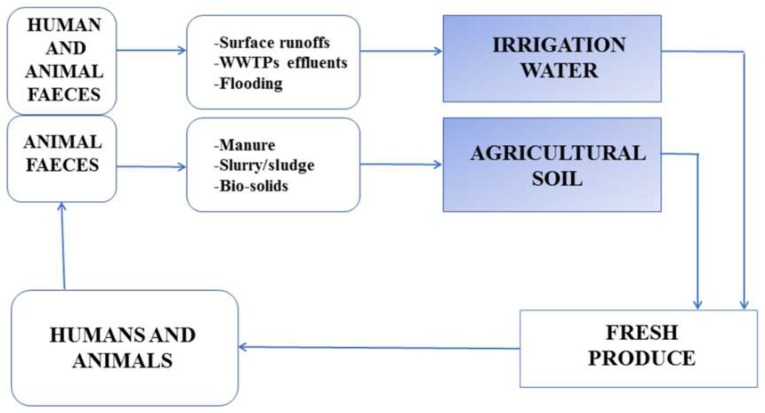
The cycle involved in the transmission of enteric pathogens from the human and animal faeces through the farm to fresh produce destined for human and animal consumption. WWTPs—wastewater treatment plants.

The contamination routes, as well as the frequency of fresh produce contamination on the farm usually varies with cultivation locations, and this is as a result of different environmental factors such as climate conditions, topography, land use interactions and proximity to animal rearing sites [88]. Generally, the primary origin of enteric pathogens on the farm is from human and animal faecal materials. In the USA, a massive outbreak of *E. coli* O157:H7 associated with spinach was traced back to the faeces of feral swine during environmental investigation using molecular typing [89]. These faecal materials directly or indirectly find their way into the water bodies used for plant irrigation usually via surface runoffs, discharge of final wastewater treatment effluents and flooding or onto agricultural soil upon which plants are grown via manure application, deposition of sludge or biological solids, thus contaminating the produce that is destined for either human or animal consumption (Figure 1). Some of these pathogens are ingested by humans or animals, in that process, producing disease conditions, especially in immuno-compromised individuals. The pathogens are again passed out along with faeces and the cycle continues. Irrigation water and manure-amended agricultural soil represent the two most important transmission pathways of enteric pathogens to fresh produce during the primary production stage of fresh produce [86].

### 4.1. Irrigation Water

Irrigation is the application of water to the soil during agricultural production of farm produce to maximize the yield of fresh produce, and this is especially done during the dry seasons [90]. Irrigation water is used to supplement inadequate rainfall in many regions of the world, and being a critical requirement for the optimum production of farm produce, it is necessary to irrigate plants whenever needed [91]. Availability of abundant sources of potable water is essential for the production of fresh produce, although, accessibility to safe water is progressively becoming a challenge worldwide resulting in increased food safety risks [13]. These challenges are further complicated by the swift rate of industrialization, urbanization, climate change and global warming. Many parts of the world are confronted with the challenges of either addressing the shortages of freshwater supplies or the prevention of pollution of the few that are readily available [92], especially in semi-arid countries like South Africa. This in extension threatens the overall yield of fresh produce, since farm production greatly depends on the availability of safe freshwater supplies. In some developed countries like England, only 1% of all water sources are used for irrigation, however, in some other high-income countries like Portugal and Spain, 70% are reserved for irrigation purposes [93]. Also, in some developing countries like India, 90% of water sources available are used for irrigation purposes [93], while in South Africa, 70% is needed for the same purpose [94]. These disparities suggest that the volume of water required for irrigation purposes goes beyond the developmental status of a nation but also depends on the climate conditions of the country [15].

#### 4.1.1. Sources of Irrigation Water

Usually, the quality of water used for irrigation is contingent on the source of the water, and the following sources of irrigation water are listed in the order of decreasing level of microbial hazard; untreated or inefficiently treated wastewater, surface waters, shallow groundwater, deep groundwater, portable or rainwater [95,96,97].

##### Wastewater

Application of wastewater weather treated, raw, inadequately treated or thinned for irrigation purposes is a common practice in rural and urban areas [98]. Water scarcity in certain regions of the world such as sub-Saharan Africa amidst other factors like too much dependability of limited water source, proximity to a water source and the availability of nutrients has forced so many farmers to resort to the use of wastewater for the irrigation of fresh produce. Generally, the quality of wastewater is usually poor containing enormous amounts of impurities such as organic materials, suspended solids and pathogens, hence the need to properly treat it before being applied to fresh produce. For instance, the bacteriological assessment of the effluents of two wastewater treatment facilities in South Africa revealed a high count of *E. coli* (3 to 1.2 × 10^5^ Colony Forming Unit (CFU)/100 mL) and *Vibrio* spp. (11 to 1.4 × 10^4^ CFU/100 mL) [99]. However, adequate treatment of wastewater is only done in a few cases because the procedure is costly and time overwhelming [98]. Studies have indicated that only about 10% of wastewater is treated adequately in growing countries [13]. Wastewater that is not adequately treated contains large numbers of pathogens [100]: According to [101], up to 7000 *Salmonella* spp., 100 *Vibrio cholerae*, 600 *Ascaris lumbricoides*, 4500 *Entamoeba histolytica*., 7000 *Shigella* spp. and 5000 Enteroviruses can be harboured in 1 L of community sewage. In Hidalgo, Mexico, Pachuca City, raw vegetables used to prepare ready to eat salads were found to be irrigated with raw sewage water and 99%, 85% and 7% of the salad samples (*n* = 130) were found to be contaminated by faecal coliforms (FC), *E. coli* and diarrheagenic *E. coli* respectively [102]. The application of untreated wastewater on the farm poses a public health hazard as it represents a transmission pathway for pathogens to fresh produce destined for human consumption. Based on this, the World Health Organization advice that the concentration of faecal coliform in wastewater intended to be used for irrigation of fresh produce must not be more than 100 CFU or Most Probable Number (MPN) of 100/mL [103]. According to the WHO guidelines for the safe use of wastewater, excreta and greywater, treated greywater can be used for agricultural purposes as it possesses some health benefits such as increase in household food security, increase in nutritional variety and increase in household income which is used to support health-promoting activities such as education and access to health care. However, it can potentially transmit infectious diseases and unwanted chemicals when not adequately managed [103].

##### Surface Water

Surface water bodies including ponds, streams, rivers and dams, are not just highly valued for agricultural purposes but also domestic and recreational purposes. Globally, they are the most widely used water for irrigation, yet are more open to microbial contamination compared to other sources [13,104]. Surface water bodies are also susceptible to contamination by urban and industrial pollutants which are made up of heavy metals, carcinogenic and organic materials harbouring pathogenic microorganisms of faecal origin which can be transferred to food web when used for agricultural activities, hence pose a serious threat to food safety and public health [105]. In a study in South Africa, *E. coli* count and FC count in a river used for irrigation of vegetables were 3.5 × 10^5^ CFU/100 mls and 1.6 × 10^6^ CFU/100mls respectively [106]. In Australia, [107] detected some genes that are suggestive of the incidence of *Salmonella*, *E. coli* and *Campylobacter jejuni* in the tidal creek and pond water used for irrigation. In Georgia, [108] detected *Salmonella* in 79.2% of water samples collected from surface water bodies. In Canada, [109] recovered *E. coli* O157:H7 and *Salmonella* from Canal and River water. The tomatoes related salmonellosis outbreak due to the *Salmonella* Newport strain was traced back to the pond water used to irrigate the tomatoes [110]. Based on this, unless properly treated, it is not recommended to use surface water sources to irrigate farm crops.

Studies have shown that climate change plays a role in the transfer, prevalence and tenacity of pathogens in agricultural water and on crops [111]. Greater intensity of rainfall encourages flooding and runoffs which extends pathogens and other organic materials to surface water bodies. [112] accessed the microbial count of *E. coli* in surface water bodies in South Africa between August to October. They observed the highest microbial count that ranged from 717 to 9100 CFU/100 mL in August, which is in Winter when rainfall is more experienced.

##### Groundwater

Groundwater is usually considered as a reliable source of water during agricultural production even when there is depletion of surface water bodies [113]. The aquifers help to protect water reserves in groundwater during drought making water always available for domestic and agricultural purposes. Also, due to the enclosed nature of the groundwater, they are usually less prone to contamination compared to surface water bodies hence highly recommended for irrigation of farm produce [113].

Nevertheless, groundwater can still be polluted by pathogenic bacteria coming from inadequately disposed materials on land, landfills, industrial and chemical pollutants, sludge, septic tanks and so on. For instance, a high mean count of *E. coli* (13.91 ± 9.16 CFU/mL), total coliforms (2166 ± 95.24 CFU/mL), *Staphylococcus* (674 ± 18.21 CFU/mL) and *Clostridium* (1368 ± 33.78 CFU/mL) was observed in water samples collected from borehole in Kenya [114]. The contamination of groundwater usually occurs at a very minimal rate, hence relatively safe microbiologically for agricultural purposes [113]. Unfortunately, climate change causes increased variations in precipitation and extreme weather conditions, leading to longer periods of droughts and floods which directly impacts the availability and accessibility of groundwater. In extended periods of droughts, there is an increased chance of aquifers depletion, particularly when the aquifers are small and shallow. Due to the buffering capacity of groundwater, climate change intensifies the use of groundwater which is almost always available. However, this poses a risk to food and water security [115].

##### Harvested Rainwater

Harvesting of rainwater is a convenient alternative source of water for irrigation especially as the demand for water continues to increase [15]. Rainwater quality can be influenced by the method of collection and this can either be done from the roof and directed into basins, tanks or containers or harvested from runoff on the ground and directed into basins, tanks or any reservoir. Rainwater harvested from the roof may be impacted by bird’s droppings or debris found on the rooftops while rainwater harvested from runoffs may be impacted by pathogens resident or flowing in the soil such as *Salmonella* spp. from animal faecal droppings. It has been shown that harvested rainwater is an invaluable source of water for irrigation and household purposes particularly in rural areas where people have limited access to pipe-borne or bore-hole water [15].

#### 4.1.2. The Microbiology of Irrigation Water

Irrigation water is a potential transmission route of fresh produce contaminants [13,14]. Sources of irrigation water are usually polluted by constant influents of faecal materials, sewage, soil and other materials capable of introducing enteric pathogens into the irrigation water [12]. About 71% of irrigation water in the UK is sourced from surface water bodies that collect sewage effluents that are treated [116]. Ref. [117] recovered *E. coli* O157:H7 from 2% of river water utilized for irrigation. Benjamin et al. (2013) detected *E. coli* O157:H7 in streams and tributaries present in leafy green agricultural sites in California [118]. Castro-Ibáñez et al. (2015) detected *Salmonella* spp. in irrigation water [119]. Other sources of irrigation water mainly the roof-top harvested rainwater is known to also harbour bacterial pathogens such as *Salmonella* spp., *Aeromonas* spp., *Listeria* spp. and *Campylobacter* spp. [120,121,122,123] as well as the conventional faecal indicator *E. coli* [124]. *Legionella* spp., *Campylobacter* spp. and *Salmonella* spp. was confirmed by [125] in roof-top harvested irrigation water, and all of this is detrimental to the safety of fresh produce which is mostly eaten without rigorous treatment. The application of irrigation water containing *Salmonella* Typhi on radish and carrot led to their contamination at harvest, with the organism surviving for about 203 days in the soil after application [126]. In another study, single irrigation of lettuce plant with water containing *E. coli* O157:H7 transferred the pathogen to the plant and noticed 30 days after inoculation, yielding increased populations when plants were further contaminated at days 7 and 14 of the study [127]. In South Africa, the bacteriological quality of the famous Msunduzi water body where water used for farm irrigation is usually sourced from was carried out and found to harbour up to 84,000 MPN/100 mL total coliforms, 7900 MPN/100 mL *E. coli* and even *Salmonella* spp. after 13 months of sampling [128]. A high count of *Enterococci* (0–5.3  ×  10^5^ CFU/100 mL), total coliforms (1.9  ×  10^2^–3.8  ×  10^7^ CFU/100 mL) and faecal coliforms (0–3.0 × 10^5^ CFU/100 mL) were observed in the Buffalo river where water for the irrigation of fresh produce is sourced from [129].

The capacity of a disease-causing microbe to reside or even persist in a particular environment is crucial for it to be able to pose a serious hazard to public health. This is simply because they are constantly present in that environment and so are available to cause repeated episodes of disease outbreaks. For example, *E. coli* O157:H7 can exist for an extended period in irrigation water sources due to its ability to withstand certain unfavourable environmental conditions hence it is the most implicated bacterial pathogen involved in fresh produce outbreaks linked to water [88]. [130] noticed that *E. coli* and *Salmonella* Typhimurium were extremely stable in groundwater with strong H_2_S odour at a pH of 7.6 and a temperature of 22 °C. Also, [131] noticed that 8 × 10^4^ CFU/mL of *E. coli* O157:H7 persisted in surface water bodies at a pH of 6.2–8.9 and a temperature of 10 °C. [132] recovered *E. coli* in groundwater samples collected throughout the year with the temperature and pH of the field being 4 °C and pH of 5.6 ± 0.3 respectively and temperature and pH of the riparian area being 20 °C and 5.4 ± 0.4. But, [133] noticed a swift reduction of *S.* Typhimurium in groundwater to levels that is not detectable in 12 days at temperature and pH of 21 °C and 7.3 respectively. This suggests that the tenacity of pathogens in irrigation water sources can be affected by certain environmental factors such as temperature and pH. Nonetheless, irrigation water continues to serve as a potential reservoir of pathogenic microbes responsible for vegetable produce outbreaks [15]. In Sweden, an outbreak of *E. coli* O157:H7 infection linked to contaminated iceberg lettuce in 2005 with about 135 cases reported were linked to the irrigation water used during primary production [134]. Also, an outbreak of enterohaemorrhagic *E. coli* linked to fresh salad in 2013 was traced back to the irrigation water as the most possible source of the outbreak by the outbreak control team in Sweden [135]. The strain of *E. coli* O157:H7 that caused an outbreak linked to pre-packed spinach in the U.S. was detected in the river where irrigation water is normally sourced from and also from faecal materials of animal origin [136]. According to the report of the traceback investigations, the irrigation water was inadvertently contaminated with *E. coli* O157:H7 in the faecal materials, which eventually caused the outbreak [137]. While irrigation water continues to serve as a potential route of fresh produce associated foodborne disease outbreaks, direct evidence in this regard still remain vague [138].

#### 4.1.3. The Effect of Irrigation Application Methods and Timing on the Microbial Contamination of Fresh Produce

The method of applying water on produce on the farm usually depend on water availability and the nature of crops involved [139]. According to Centres for Disease Control and Prevention (CDC), the common types of irrigation application methods include (i) surface irrigation method in which water is circulated throughout the land without using any pump rather by gravity, (ii) localized irrigation method in which water is circulated throughout the farm using networks of pumps attached to each plant under low pressure, drip irrigation method where by water is distributed in drops to areas that is very close to the roots of plants in such a way that runoff and evaporation of water is reduced, (iii) sprinkler irrigation method where by water is circulated in the field under high pressure using a fixed or moving overhead sprinklers, (iv) centre-pivot irrigation method where by water is distributed on the farm using towers of sprinklers that have wheels and is able to move in a circular fashion, (v) lateral move irrigation method which involves the use of labour or a built machine to rotate several wheeled pipes having a set of sprinklers across the farm, (vi) sub-irrigation method in which water is dispersed over lands with high water tables using canals, ditches, pumping stations and gates, and finally (vii) the manual irrigation method where water is applied to lands using manual watering cans [140].

The contamination tendencies of the edible parts of farm produce can be influenced by the method of irrigation [4]. The sprinkler irrigation method has been shown to possess higher risks of farm produce contamination compared to surface or drip irrigation [141]. A laboratory and greenhouse research showed that *E. coli* O157:H7 persisted on the leaves of lettuce for 20 days after spray irrigation with contaminated water and the concentration of the pathogen increased with continuous irrigation [142,143]. Another study also showed a correlation between increased levels of enteric pathogens on butterhead lettuce with overhead sprinkler irrigation method [144]. [145] noticed that repeated irrigation of water by spray method containing a low dose of *E. coli* O157:H7 (about 3.5 log CFU/mL) caused an internalization of the microbes in the leaves of parsley and spinach but did not in lettuce and this pathogen remained on the leaves of the plant after 2 days of irrigation suggesting that harvesting of farm produce shortly after irrigation increases microbial risks associated with farm contamination. This shows that there is an intricate relationship between the timing of irrigation and microbial contamination of farm produce. Also, [146] showed in their study that the probability of contaminating farm produce by enteric bacteria from irrigation water is more when irrigation is done at night and during winter periods.

Some mechanisms of minimizing fresh produce contamination by irrigation water have been proposed and these include minimizing the influx of other water sources such as runoffs, lateral movement of water on sub-soil, discharge of sewage into the main source of water destined for irrigation as well as minimizing the influx of pathogens from possible microbial reservoirs such as the bottom of water tanks, algae and periphyton [147]. Also, the treatment of water using cost-effective techniques like filtration, coagulation, disinfection, flocculation and even irradiation while the water is in storage, between storage and discharge systems and in discharge systems is another feasible strategy to guarantee the microbial quality of irrigation water. Other techniques like the ultrasound and ultraviolet C (UV-C) have been found to be effective as well, possessing advantages like high bactericidal action, not affected by pH, easy to use, no formation of disinfection by-products and low operational costs [104]. Improving the bacteriological quality of surface and wastewaters using sand filtration techniques and using appropriate irrigation plans with simultaneous use of waters with varying qualities can aid in minimizing the risks of fresh produce contamination [147]. [148,149] also suggest that changing the methods of irrigation may influence the availability of pathogens to plants. The quality of water used for irrigation should be considered when making a choice on the application method especially when the plants are due for harvesting. For example, the spray irrigation method increases the chances of plant contamination while well-maintained hydroponic systems, drip and furrow irrigation method do not [147]. This informs growers using water sources of poor microbiological quality to use the application methods that avoid contact between the edible part of the crop and the irrigation water.

### 4.2. Agricultural Soil

The agricultural soil may naturally contain certain pathogens such as *Listeria* spp. or receive them during soil amendment using animal manure and this might directly contaminate the food crops that are grown on it through the splashing of soil particles by heavy rainfall or sprinklers onto the edible parts of the plants [12]. The fact that so many agricultural soils are constantly open to direct and indirect sources of microbial contaminants including microbial tinged irrigation water, animal dung, free-ranging animals, run-offs, municipal sewage and effluents makes them a receptacle for numerous pathogens [88]. Usually, the incidence, existence and persistence of pathogenic bacteria in the agricultural soil ecosystem is contingent on several factors such as the nature of the soil, level of acidity and alkalinity of the soil, moisture level of the soil, temperature, presence of organic materials, soil biotic connections and cultivational activities carried out on the soil. For instance, the average survival of *E. coli* O157:H7 and *Salmonella* spp. in the soil is 7 to 25 weeks but this is contingent on the type of soil, water level of the soil, temperature and the origin of contamination [150,151,152,153]. Evidence has shown that certain zoonotic bacteria such as *Salmonella* spp. [154] persist for extended periods in clay soil that is moist and at low temperature [8]. Also, evidence has shown an increased level of pathogens in manured soils compared to non-manured soils especially sandy soils which possess much lower water holding capacity [155,156,157,158]. Although, [159] observed in his experiment that *Salmonella* Typhimurium thrived more in sandy soil than in clay soil and this is correlated to the fact that the sandy soil was more alkaline and contained more organic materials. [160] noticed a rapid reduction in the amount of *Salmonella* Typhimurium in both silty-clay and sandy-loam soil after the application of human urine to the soil as opposed to after the application of cow dung. [161] noticed that *E. coli* O57:H7 survived between 21 and 45 days in fallow soils after the application of dairy manure. [162] noticed that the application of poultry waste better favoured the existence of *E. coli* O157:H7 in silty-loam, sandy-loam and clay-loam soils compared to horse dung. In the experiment of [163] 50% moisture at 30 °C with pH of 8.74 ± 0.04, 5.97 ± 0.03, 8.42 ± 0.04/6.05 ± 0.01 and carbon content of 3454.8 ± 32.6, 9957.0 ± 280.3, 5744.4 ± 628.1/7968.9 ± 576.4 favoured the growth and existence of *E. coli* O157:H7 in clay-loam, clay and loamy soils respectively.

Soil water levels are usually impacted by the level of precipitation and irrigation and this influences the occurrence and dissemination of pathogens within the soil ecosystem. It has been shown that rainwater induced flooded soil rich in organic contents supported the survival of *E. coli* [158,164,165]. The pH of the soil influences the biogeochemical pathways mediated by soil resident bacteria hence impacting their diversity within the soil environment [88]. It has been shown that neutral pH is the ideal pH for the existence of bacteria in the soil [166]. The prevalence of bacteria in the soil is also affected by the type of soil and these variations are based on the extent of organic content of the soil, the size of soil particles, porosity and water retention ability of the soil [167,168]. Evidence has shown that *E. coli* can exist in clay and loamy soil for up to 25 weeks but can barely exist in sandy soil for 8 weeks [152]. Generally, bacterial cells are easily adsorbed to the particles of clay soil compared to other soil types making them not only more tenacious but also protected from other predatory and parasitic microbes [169]. In one instance where the cells of *E. coli* were strongly adsorbed to the clay soil, their life span was prolonged because they were protected from the toxic effects of protozoa [170]. Another factor that influences the distribution of bacteria in the soil is temperature. Generally, low soil temperatures favour the growth of bacteria in the soil. Also, the availability of organic materials in the soil encourages the growth and survival of resident bacteria as this act as nutrient and carbon source and help to retain moisture in the soil [171]. Certain agricultural practices also determine the existence and tenacity of bacterial pathogens in the soil. The improvement of soil fertility using either organic or inorganic fertilizers encourages the existence of pathogens in the soil by supplying them with necessary nutrients such as phosphorus, sulphur, nitrogen, potassium, magnesium and calcium and required for their growth [172]. These nutrients also increase the pH of the soil in favour of the growth of some of the soil pathogens [162,173].

### 4.3. Manure

It is routine in many countries to apply animal dung onto agricultural soil during primary production of food crops mainly vegetables and fruits with the intention of improving the fertility of the soil, however this practice increases the transferability of enteric pathogens to farm produce especially when the animal waste, slurry or manure compost are not properly treated [174]. There is a high correlation between the presence of organic matters in the soil and the incidence of enteric pathogenic bacteria in the soil as well as farm produce grown on the soil. In addition, the presence of animal dung on the soil increases the capability of bacterial pathogens to thrive in the soil for several months and years, thus causing preharvest safety issues. Manure amendment of soil usually involves the even application of waste materials which could either be solid, semi-solid or liquid originating from domestic or wild animals on agrarian soil [4]. Several pathogenic bacteria have the tendency to persist in the manure for length of time, however their survival depends on certain factors ranging from the physicochemical properties such as moisture content and pH to the source of the manure, manure treatment procedure, aeration, type of soil destined to be amended and the extent of manure application [175,176]. [177] noticed that *E. coli* O157:H7 thrived longer in farmyard manure and slurry under anaerobic conditions. [178] noticed an experimental decline in the concentration of *Salmonella* Infantis in fresh slurry exposed to aeration. [179] noticed that temperature levels of 64 °C to 67 °C reduced the concentration of *Salmonella* spp. during window composting. [180] noticed that no viable cells of *Salmonella* Newport remained in the sewage sludge that was composted for 43 h at 60 °C. [181] noticed a swift decline in *Listeria monocytogenes* viable cells in cattle slurry at 17 °C as compared to 4 °C. [182] observed that initial moisture levels of 30% increased the concentration of *Listeria monocytogenes* in dairy compost. The makeup of manure is influenced to a large extent by the animal feed composition and this also determines the kind of pathogens that will occur in the manure as well as their capacity to survive treatment procedures [183]. The incidence of some pathogens such as *Salmonella* spp. and *E. coli* O157:H7 in cattle have been proposed to be influenced by the nature of diet of the cattle as a diet high in energy and poor in fibres favours the survival of these pathogens in the manure originating from these animals [184]. Notwithstanding, several methods are available to treat manure prior to application on the soil such as composting which ordinarily, is sufficient to terminate bacteria at 55 °C for 3 days, pelleting which is highly recommended when treating poultry manure, conditioning, alkaline stabilization, dry heating etc. [88]. There have been drawbacks with regards to the efficiency of some of these techniques. For instance, there have been reports on the resuscitation of bacteria in a cooled heat-induced compost [175,185]. Therefore, several regulatory bodies advise that a good amount of time should be left between when manure is applied and when farm produce is harvested to prevent possible contamination of the produce. The USDA National Organic Program Regulation advised that at least 120 days should be left amid the application of untreated manure and the harvesting of farm produce whose edible portions are in contact with the soil and a minimum of 90 days for farm produce whose edible portions are not in contact with the soil [186].

### 4.4. Animal Intrusion

The intentional or unintentional intrusion of either domestic or wild animals into agricultural sites can lead to preharvest contamination of farm produce [86]. The contamination of fresh produce has been sourced tracked to animals during outbreak investigations which either involved wondering wild animals intruding farm sites or cross-contamination by faeces coming from a close animal farm. Unfortunately, many of these feral animals harbour certain zoonotic diseases which are of great significance to humans [187]. Also, some of these wandering animals harbour certain bacteria pathogens such as *Campylobacter* spp. and *E. coli* O157:H7 and are able to disperse them in the agricultural milieu. In Finland, the contamination of apples in cider orchards by *E. coli* O157:H7 and *Cryptosporidium* was suspected to have come from cattle or deer [188], that of strawberries by *E. coli* O157:H7 was suspected to have come from deer [189], and that of lettuces by *Yersinia pseudotuberculosis* in Finland was suspected to have come from wild animals [190].

## 5. Some Bacterial Pathogens with Outbreak Potentials and Their Antimicrobial Resistance Trends

### 5.1. Escherichia coli

*Escherichia coli* was first described as “*Bacterium coli commune* by Theodor Escherich in 1885”, which he isolated from the faeces of new-borns [191]. This microbe is a normal microflora of the human gut and regarded as a commensal [192]. This bacteria is a member of the Enterobacteriaceae and can thrive in the absence or presence of oxygen [193]. The existence of *E. coli* in water is suggestive of recent faecal presence and possible incidence of water-borne diseases which could be a serious threat to health [194]. *E. coli* has the ability to thrive in open environments particularly the soil, manure and irrigation water and can even be transferred to farm produce such as radish and lettuce via splashing of soil particles during irrigation or rainfall, irrigation with contaminated water or the migration of the pathogen from the soil into the inner compartments of plant, hence constitution food safety problems [195]. *E. coli* can be used to carry out antibiotic surveillance because the bacterium can easily acquire resistance [196]. As a genetically diverse group, several strains of *Escherichia coli* are harmless commensals of mammals, but others have the capacity to cause either intestinal or extraintestinal disease such as nosocomial septicaemia, neonatal meningitis, surgical site infections, haemolytic uremic syndrome and urinary tract infections [197]. Centers for Disease Control and Prevention estimates that 265,000 Shiga toxin *E. coli* infections occur each year in the United States [198]. Recently, there are at least eight documented *E. coli* pathotypes and at least six groups causes diseases of the gastrointestinal tract and are the main cause of diarrhoeal diseases in low-income countries namely; Enteropathogenic *E.coli* (EPEC), Enteroaggregative *E.coli* (EaggEC), Enteroinvasive *E.coli* (EIEC), Enterohaemorrhagic *E.coli* (EHEC), Enterotoxigenic *E.coli* (ETEC), Shiga toxin-producing *E.coli* (STEC) and Diffusely adherent *E.coli* (DAEC) [199]. Extraintestinal pathotypes include Necrotizing factor producing *E.coli* (NTEC) and Uropathogenic *E.coli* (UPEC) and are known to cause extraintestinal infections [200]. The incidence of antimicrobial resistance (AMR) in *E. coli* poses a great risk as they are the most common Gram-negative bacteria found in humans [201]. Treatment of ailments caused by this bacteria is complicated with the evolution of AMR to first-line antibiotics including aminoglycosides, polymixins, fluoroquinolones and even worst due to the rapid spread of extended-spectrum beta-lactamases (ESBLs) which promotes resistance to broad-spectrum penicillins, cephalosporins and carbapenems [202]. Evidence has shown that *E. coli* from animal sources also show resistance to previously active agents such as phenicols, fosfomycin, trimethoprim, tetracyclines and phenicols.

#### *E. coli* O157: H7

*E. coli* O157:H7 is a public health significant foodborne and waterborne zoonotic pathogen causing diseases in humans which range from uncomplicated diarrhoea to haemorrhagic colitis (HC) and haemolytic uremic syndrome (HUS) [203]. *E. coli* O157:H7 causes about 73,000 illnesses in the United States annually [204]. Three hundred and fifty outbreaks caused by this bacteria between 1982 and 2002 were reported to CDC, out of which 52% was transmitted through foods, 14% is from person to person, 9% through water, 3% through animal contact and 21% transmission route are unknown [204]. *E. coli* O157:H7 either harbours one or two toxins of which one is counteracted by Shiga toxin antisera extruded by *Shigella dysenteriae* type 1 known as Shiga toxin 1 (Stx1) and the other Shiga toxin 2 (Stx2), not counteracted by these antisera [205]. Stx1 is further subdivided into Stx1a, Stx1c and Stx1d subtypes while Stx2 is divided into Stx2a, Stx2b, Stx2c, Stx2d, Stx2e, Stx2f and Stx2g subtypes [206]. Production of these virulence factors happens to be the hallmark for the pathogenicity of this bacteria. This bacteria also extrudes somatic (O) antigen 157 and flagella (H) antigen 7 [207] and just as other serotypes of *E. coli,* they ferment lactose, however, they have a delayed D-sorbitol fermentation capability usually above 24 h and are unable to extrude β-glucuronidase, which can hydrolyse 4-methyl-umbelliferyl-D-glucuronide (MUG) [207]. Because of this, MUG supplemented Sorbitol MacConkey (SMAC) agar is the media of choice for the culture of this bacteria. In some cases, potassium tellurite, vancomycin or cefixime is added to increase the selectivity of the media by killing non-targeted bacteria present [207]. *E. coli* O157:H7 does not thrive optimally between 44–45.5 °C, which is the normal temperature used for the isolation of *E. coli* from aquatic and food samples [205]. Cattle are established carriers of *E. coli* O157:H7 and they do this asymptomatically [208], because they lack the receptors for Shiga toxins in their vascular system [209]. Other animals these bacteria have been recovered from include dogs [210], sheep [211], deer [212], goats [213] and horses [214]. The faecal materials of these animals are either intentionally applied on agricultural soils to improve its fertility or they are unintentionally dispersed to soils and nearby surface water bodies via runoff. Fruits and vegetables reserved for human eating get contaminated with *E. coli* O157:H7 via contact with animal faecal materials when they are cultivated on soil amended with untreated animal manure containing this bacteria or by irrigation of vegetables with water contaminated with sewage or animal faecal materials [215]. Also, the contamination of farm produce can ensue via the transportation of *E. coli* O157:H7 from manure-amended soil, through plant roots into the comestible parts of farm produce [127]. Management of infections caused by this bacteria does not really require administration of antibiotics due to the increased chances of HUS development, however, it is proposed that the cell wall and protein inhibitors be administered when certain information regarding the serotype, virulence factors, duration of disease and immune status of patients are known [216]. Notwithstanding, AMR in *E. coli* O157:H7 have been documented and this may be caused by the indiscriminate use of antibiotics in animal husbandry [206]. STEC *E. coli* O157:H7 and non O157 isolated from farmstead animals in North-Western Mexico were shown to exhibit resistance to ampicillin, cephalothin, chloramphenicol and kanamycin [217]. Also, *E. coli* O157:H7 recovered from sheep and cattle exhibited resistance to cephalothin, streptomycin, nalidixic acid, sulphamethoxazole, sulphonamide and streptomycin [218]. In South Africa, *E. coli* O157:H7 recovered from dairy cattle faeces showed resistance to cephalothin, ampicillin, cefuroxime, amoxicillin/clavulanate and ceftazidime harbouring the *bla*_ampC_, *bla*_CMY_, *bla*_CTX-M_, *bla*_TEM_, *tet*A and *str*A resistance genes [206].

### 5.2. Salmonella spp.

*Salmonella* genus is made up of Gram-negative, flagellated, rod-shaped, facultative anaerobes belonging to Enterobacteriaceae [219]. The genus consists of two main species; *Salmonella enterica* and *Salmonella bongori* which again is composed of more than 2500 recognized serotypes [220]. The human disease-causing Salmonellae are typically grouped into a small number of invasive typhoidal serotypes (*Salmonella enterica serovars Typhi*, *Paratyphi A*, *Paratyphi B*, and *Paratyphi C*) which are restricted to humans causing typhoid fever and non-typhoid fever both denoted as enteric fever, and thousands of non-typhoidal *Salmonella* serotypes regarded as NTS serotypes, which have wide vertebrate host range, including nonhuman animal species producing diseases such as diarrhoea [221,222]. Both typhoidal and non-typhoidal invasive *Salmonella* infections are the major cause of mortality and morbidity especially in developing regions such as Sub-Saharan Africa, parts of India and Asian sub-continent, with inadequate sanitation and limited access to safe food and water [223,224]. Globally, *Salmonella* spp. is the chief etiologic agent of food-related outbreaks [225,226]. It is projected that above 94 million gastroenteritis cases and 155,000 mortalities are attributed to *Salmonella* every year and 85% of these are food inclined [227,228]. Recently, food-related outbreaks of salmonellosis are increasingly associated with farm produce and fruits. In some cases, the higher incidence is attributed to vegetables than other food products [229]. This shows that environmental transmission of this pathogen can cause human infection, and so recent epidemiological studies have focused on the possible preharvest produce contamination routes including irrigation water, agricultural soil and manure [97,108,230,231,232]. Cases of salmonellosis are increasingly linked to the consumption of farm produce contaminated by irrigation water [233], resulting in a number of clinical syndromes such as typhoid fever, gastroenteritis, bacteraemia and focal infections [219]. In this case, antimicrobial therapy remains the only option available to salvage this formidable public health challenge, however, *Salmonella* species continue to exhibit multidrug resistance making it difficult to treat patients with severe infections [234]. *Salmonella enterica* has shown resistance to traditional first-line antibiotics including chloramphenicol, ampicillin, trimethoprim-sulfamethoxazole and fluoroquinolone. In the same vein, typhoidal and non-typhoidal *Salmonella* strains have also shown resistance to Extended-spectrum cephalosporins, however, azithromycin is still potent for the treatment of uncomplicated typhoid fevers and could be used as a substitute in areas with high fluoroquinolone resistance [223].

### 5.3. Shigella spp.

*Shigella* spp. is among the main infectious food contaminants that can cause illness even at a low dose of infection [1]. They possess numerous virulence determinants that contribute to their colonization and invasion of the epithelial cells which subsequently causes the termination of the host cells, starting with the entry of the bacterium into the cells of the epithelium, intracellular growth, inter-cellular spread and eventually death of the host cell [235,236]. *Shigella* spp. causes about 500,000 cases of diarrhoea in the United States annually [225]. Usually, four species of *Shigella* including *Shigella flexneri*, *Shigella dysenteriae*, *Shigella sonnei* and *Shigella boydii* are frequently responsible for diarrheal disease and are sub-grouped into groups A-D respectively [237]. *Shigella* spp. remain the etiologic agent of the severe food-borne shigellosis which is an acute enteric infection, clinically manifested by dysentery [238]. Shigellosis is a severe population health problem that instigates morbidity and mortality in both developed and undeveloped countries [237]. Approximately 1.1 million people die as a result of shigellosis while 164.7 million people get afflicted by diarrheal disease caused by *Shigella* spp. every year [236]. *S. flexneri* is the main cause of endemic shigellosis especially in growing countries while *S. sonnei* is more rapidly isolated in developed countries [239]. However, with recent epidemiologic findings in developing countries, serotypes of *S. flexneri* have been substituted by *S. sonnie* due to economic growth and advancements in hygiene [240]. Foods especially fresh fruits, vegetables and unpasteurized milk serves as important transmission routes of *Shigella* spp. and their antibiotic-resistant strains responsible for human cases of shigellosis [238]. The evolution of antibiotic-resistant strains of *Shigella* poses a great challenge to the physicians during shigellosis treatment [238]; hence it is critical to comprehend the antibiotic resistance pattern of this organism [241]. Resistance to trimethoprim/sulfamethoxazole (TMP/SMX) as well as multidrug resistance (MDR) has been increasingly observed in *Shigella* spp. Eighty-nine percent of *S. sonnei* strains involved in the MDR infection outbreak in the USA in 2005 showed resistance to ampicillin and TMP/SMX [242].

### 5.4. Klebsiella spp.

*Klebsiella* genus belongs to the tribe Klebsiellae which is a member of the group Enterobacteriaceae [243]. *Klebsiella* was named after a German microbiologist, Edwin Klebs in the 19th century. These organisms are rod-shaped, non-locomotive, Gram-negative bacteria composed of polysaccharide capsule which covers the entire surface of the cell protects the cell from adverse host defence mechanisms and gives the bacteria a characteristic appearance in Gram stain [243]. *Klebsiella* spp. are ubiquitous, though found majorly in either the environment including surface water bodies, soil, manure, sewage and plants or on the surfaces of mucous membranes of humans and animals such as horses, pigs etc. [244].

The environmental and clinical isolates of *Klebsiella* spp. are almost indistinguishable in terms of virulence and biochemical properties, readily causing diseases like soft tissue infections, meningitis, pneumonia, diarrhoea, septicaemia and urinary tract infections [244,245]. In humans, most of the above-mentioned infections are caused by *Klebsiella pneumoniae* which is followed by *Klebsiella oxytoca,* particularly among the immuno-compromised. *Klebsiella* spp. are usually resistant to multiple drugs with plasmids being the primary source of resistance determinants [246]. In the past, they have shown resistance to fluoroquinolones, chloramphenicol, tetracyclines, trimethoprim/sulfamethoxazole and aminoglycosides [247]. They also have the ability to produce ESBLs, enzymes which have made them resistant to virtually all beta-lactam antibiotics including carbapenems. Ever since ESBLs emerged, *Klebsiella* spp. are more frequently implicated in outbreaks caused by multidrug-resistant Gram-negative bacteria (MDR-GNB), and so ESBL-producing *Klebsiella* spp. are considered a menace in clinical medicine [248].

### 5.5. Citrobacter spp.

*Citrobacter* spp. are coliforms belonging to Enterobacteriaceae that normally resides within the gut of animals and humans. They exist in the milieu particularly the soil, sewage and water bodies indicating potential water contamination [249,250]. This means that *Citrobacter* spp. have the ability to be transferred from the farm to fresh produce destined for consumption by humans particularly via irrigation water and soil since they are residents of the soil and water, thereby constituting a nuisance to public health. In humans, they are considered as opportunistic pathogens causing ailments such as wound infections, urinary tract infections (UTIs), septicaemia, pneumonia, endocarditis, gastroenteritis, brain abscesses and meningitis especially among children and the immunocompromised, thus having a high mortality rate [251]. *Citrobacter* genus is made up of over 11 genomospecies distinguishable by their biochemical characteristics [252]. *Citrobacter freundii* is the most common infection causing species of this genus followed by *C. youngae* and *C. braakii* which barely produce infections in humans. *Citrobacter freundii* usually acquire virulence factors like cholera toxin B subunit homolog, Shiga-like toxins and heat-stable toxins [250], making them cause food poisoning and diarrhoea in humans [253]. Certain *Citrobacter* spp. exhibit resistance to some antibiotics because they harbour plasmid-encoded resistance genes. Some *C. freundii* strains possess inducible *ampC* genes encoding resistance to ampicillin and first-generation cephalosporins. ESBLs in *Citrobacter* have also been documented, especially the SHV, TEM and CTX−M types [254].

### 5.6. Enterobacter spp.

*Enterobacter* spp., members of Enterobacteriaceae are Gram-negative, motile, facultatively anaerobic, rod-shaped and non-sporulating bacteria. Though this group of bacteria falls under the coliform bacteria, they are not specifically considered as faecal coliforms due to their inability to thrive at 44.5 °C in the presence of bile salts just like *E. coli* [255,256]. *Enterobacter* spp. are pervasive in the environment including the soil, water and sewage in association with plants and food materials and this are related to the fact they are constantly present in the gut of humans and animals [257]. Numerous species of these bacteria including *E. gergoviae*, *E. agglomerans*, *E. cloacae* and *E. aerogenes* are pathogenic, causing opportunistic infections especially among the immunocompromised including lower respiratory tract infections, bacteraemia, urinary tract infections endocarditis, septic arthritis, soft-tissue infections, intra-abdominal infections, ophthalmic infections and infection of the central nervous system. *Enterobacter cloacae* and *Enterobacter aerogenes* are the two most clinically significant species [258]. The emergence of antimicrobial resistance in *Enterobacter* spp. has tremendously affected treatment options especially in nosocomial settings where these organisms thrive. Treatment of *Enterobacter* infections commonly adopts a combination therapy which involves multiple antibiotics with different parent structures, for example, a combination of an aminoglycoside or a fluoroquinolone with a beta-lactam. This looks promising; however, it also leads to the selection of multidrug-resistant pathogens [259].

### 5.7. ESBL-Producing Enterobacteriaceae

Generally, ESBLs are a group of enzymes that inhibits the effects of most beta-lactam antibiotics, including penicillins, cephalosporins, monobactam and aztreonam, thus rendering them ineffective [260], with CTX-M representing the most common ESBL genetic variant [261,262]. The major types of ESBLs include the SHV-type which appears to be derived from *Klebsiella* spp., TEM-type (TEM-1 was first reported from an *E. coli* isolate in 1965), CTX-type, which is a new family that selectively cleaves cefotaxime and is commonly found in *E. coli*, *Salmonella* Typhi and other members of Enterobacteriaceae, PER-type (PER-1 hydrolyses penicillins and cephalosporins although is susceptible to clavulanic acid inhibition), GES-type which was initially defined in a *K. pneumoniae* isolate from newborn patient in France, as well as other ESBLs such as VEB-1, BES-1, CME-1, SFO-1 and GES-1 which are sparingly reported [260]. ESBL production is considered as the most important antibacterial resistance mechanism in Enterobacteriaceae obstructing the efficacy of most available antibiotics during treatment of infections [260]. They have been isolated from the lung, abscesses, catheter tips, sputum, throat culture and blood peritoneal fluid [263]. Beta-lactam antibiotics are usually utilized for the remediation of ailments produced by Gram-negative bacteria, and so constant exposure of these group of bacteria to numerous β-lactams induces continuous development of ESBLs [260]. ESBL Enterobacteriaceae (ESBL-Eb) are opportunistic pathogens which are generally found in the human and animal gut microbiota, causing infections especially among the immunocompromised, geriatrics and paediatrics [264]. ESBL-Eb harbours a wide range of beta-lactamase enzymes that allows them to develop resistance to a wide array of penicillins, early and extended-spectrum cephalosporins, aztreonam and recently to cephamycins and carbapenems [265,266]. Table 2 summarizes the common types of β-lactamases that occurs in Enterobacteriaceae.

### 5.8. Listeria Monocytogenes

*Listeria monocytogenes*, one out of the 15 taxonomical species of the genus *Listeria* [268], is a Gram-positive, ubiquitous, intracellular, non-sporulating, rod-like, motile and a facultative anaerobe which is broadly dispersed in the agricultural milieu including soil, water and manure [269,270,271]. In the soil, they exist as saprophytes but becomes pathogenic once they get into animal and human cells [272]. Other known reservoirs of *Listeria monocytogenes* include plant materials, vegetation, farms as well as infected humans and animals which intermittently pass out *Listeria monocytogenes* present in their gut [273]. Although *Listeria monocytogenes* is a foodborne pathogen, causing contamination of processed ready-to-eat foods such as sausages, raw milk products, deli meat or smoked fish [274], several studies have also linked listeriosis outbreak to fresh produce, including raw and minimally processed vegetables, and this trend will continue to manifest as long as this pathogen is present in the growing environment particularly the water and soil [272]. *Listeria monocytogenes* are adaptable to adverse conditions including wide temperature range (−0.4 °C to 45 °C), pH (4.0 to 9.6), water activity (above 0.90) and can thrive under aerobic and anaerobic conditions, however cooking temperature of 65 °C and above can kill *Listeria monocytogenes* [273]. *Listeria monocytogenes* can persist on the surfaces of food processing materials and facilities for several months and years as biofilms, which can tolerate high amounts of environmental agents including sanitizers, disinfectants and antimicrobials [275]. Thirteen serotypes of *Listeria monocytogenes* have been identified, however, only serotype 1/2a, 1/2b, 1/2c and 4b causes more than 95% of human listeriosis [276]. This disease is rare with about 0.1 to 10 cases per 1 million people per year depending on the countries and regions of the world [277], but highly dangerous, producing two major types of disease conditions; (i) invasive listeriosis, whereby the pathogen causes infection in the delicate parts of the body including the spleen, liver, cerebral spinal fluid and blood-producing signs and symptoms ranging from diarrhea and fever in healthy adults to diarrhea, fever, abortion and miscarriage in prenatal women and sepsis, meningitis and pneumonia in neonates (ii) non-invasive listeriosis, whereby the pathogen causes non febrile gastroenteritis [272]. *Listeria monocytogenes* has the highest mortality rate among the foodborne pathogens targeting mostly the foetus, paediatrics, pregnant women and immunocompromised adults [278].

The largest outbreak caused by this pathogen was experienced in South Africa between 1 January, 2017 and 14 March, 2018 where about 978 laboratory-confirmed cases of listeriosis were reported to the National Institute of Communicable Diseases (NICD) [279]. The case fertility rate was 183 and comprised majorly of neonates, elderly, immunocompromised adults and pregnant women. Whole-genome sequencing of the isolates recovered from the patients showed that 91% of the strains belonged to the sequence type 6 (ST6) which was also detected in polony, a ready to eat meat product as well as in the processing environment, hence believed to be the source of the outbreak [279]. Other strains may have been involved in the outbreak as indicated in the situation report prepared by the National Listeria Incident Management Team [280]. 

Antibiotics generally used against Gram-positive pathogens are very active on *Listeria monocytogenes* with beta-lactams like ampicillin been the drug of choice, usually administered alone or in combination with gentamicin [269]. In situations where allergy to beta-lactams is observed, vancomycin, erythromycin, trimethoprim/sulfamethoxazole and fluoroquinolones are used as alternatives [269]. *Listeria monocytogenes* are recently shown to acquire resistance to antibiotics like cefepime, oxacillin, lincosamides, cefotaxime and fosfomycin [281], and this is influenced by the way antibiotics are used and the diversity of geographical locations [282]. It is therefore paramount to constantly monitor the antimicrobial resistance pattern in *Listeria monocytogenes* within different geographic locations, to determine the right set of antibiotics that are still useful for the treatment of listeriosis within that region.

## 6. The Impact of Antibiotic Resistance

Infections due to the ingestion of contaminated fresh produce are mostly caused by specific strains of bacteria like *Salmonella* spp., *Listeria monocytogenes*, *E. coli* and *Shigella* spp. The contamination can occur either on the farm or later through cross-contamination. Beyond causing diarrhoeal disease, fresh produce associated infections can even lead to death. So, infections caused by these pathogens definitely require effective antibiotics for the proper treatment of patients. Unfortunately, most of the available antibiotics are losing their efficacy due to the development of antibiotic resistance in most pathogenic bacteria. This, therefore, causes extended hospital stays, increase in health care cost, and economic burden on both families and societies, thereby posing a serious threat to public health. Prudent use of antibiotics at both individual and society level will go a long way in curbing this menace [283,284].

## 7. Interventions to Prevent Fresh Produce Contamination Prior to Harvesting

One of the strategies required to minimize the preharvest dissemination of infectious bacteria to fresh produce is the stoppage of all forms of soil amendment and to some extent irrigation for some time prior to harvesting of farm produce, since the occurrence of foodborne pathogens on fresh produce via these routes is almost inevitable [285,286,287]. This formed the “90 to 120 days rule” of not harvesting farm produce 90 days (for farm produce whose edible parts touch the soil) or 120 days (for farm produce whose edible parts do not touch the soil) after the application of manure [288,289].

Also, it is recommended to cease irrigation of farm produce at least 2 to 7 days before the farm produce are harvested [290]. This is because irrigation water- and agricultural soil-induced bacterial pathogens existing on the plant surfaces will likely die off during these wait times, thereby reducing the level of food safety risks [85]. Certain laboratory experiments have been carried out to determine the efficacy of these waiting times and it was observed that the number of infectious bacteria such as *E. coli* O157:H7 and *Salmonella* spp. occurring on farm produce significantly reduced between 1 to 10 days of stoppage of irrigation and soil amendment [145,285,286,291,292,293]. Although some studies stated that some of the pathogens do not really die off but get induced into a dormant form, a state where the pathogens are viable but not culturable (VBNC) under stressful conditions [294], but returns to viable cells when favourable conditions return. These dormant cells usually possess strong resistance against any form antimicrobial agents including sanitizers and other disinfectants used during post-harvest disinfection of vegetables and fruits thus making them hard to kill [295].

Another intervention required to minimize preharvest fresh produce contamination involves the implementation of good agricultural practices (GAP) such as routine microbial testing of sources of irrigation water, proper treatment of organic fertilizers and manure before application onto agrarian soil, allowing an ample time between manure application and harvesting of farm produce, setting up of fences around the farms and irrigation water sources to prevent animal intrusion, use of appropriate irrigation method such as surface irrigation rather than spray irrigation method and regular cleaning of farm equipment [296,297].

Other preventions and mitigation strategies required to reduce the contamination of fresh produce at the preharvest level include: (i) the treatment of water during storage, between storage and delivery systems, and while in the delivery systems., (ii) development of risk assessment to identify potential point and nonpoint sources of bacterial pathogens., (iii) installation of physical barriers such as embankments, diversion dikes and berms, vegetative buffers, and ditches to re-direct or reduce runoff from animal production or waste management operations [147]., (iv) education of fresh produce growers to improve their knowledge on food safety and GAPs. and (v) outreach programs to meet the needs of these growers so as to motivate them to comply with production standards and maintain the cycle of food safety. Unfortunately, all of these do not go without certain challenges such as the inability of fresh produce growers to control adjacent land activities like animal production which directly or indirectly impacts on the safety of fresh produce via animals, run-offs, bio-aerosols or vectors such as birds, rodents and flies., and the persistence and propagation of pathogens during primary production, harvesting and transportation of fresh produce.

## 8. Conclusions

One of the strategies of acquiring good health is the consumption of a large number of assorted vegetables and fruits because it adds nutrients to our diet and protect us from several non-infectious diseases such as obesity, cancer, stroke and cardiovascular diseases. However, vegetables and fruits can serve as a reservoir of certain infectious disease agents such as *Salmonella* spp., pathogenic *E. coli* and *Listeria monocytogenes* which can cause outbreaks of infectious diseases. These pathogens are recently becoming resistant to almost all the available antibiotics thus making treatment of infections even more difficult. Despite several efforts being made to avoid the incidence of these pathogens on fresh produce, it is almost impossible to achieve it because these food products are grown on open environments where they are constantly open to different contamination sources during primary production. Amongst this, irrigation water and agricultural soil serve as two important transmission routes of foodborne pathogens to fresh produce at the preharvest level. This is because these sources do not only serve as the major receptacle of environmental materials such as effluents of WWTPs, flooding, leaching, sewage sludge, manure, and slurry but also serve as a direct link to fresh produce since they are always in contact with fresh produce during primary production, hence pose threats to food safety and human health. We, therefore, conclude that the primary production niches of the agro-ecosystem, particularly the irrigation water and agricultural soil contributes to the dissemination of fresh produce associated bacterial pathogens capable of causing an outbreak, thus increasing the global burden of diseases. Implementation of good agricultural practices on the farm will go a long way in minimizing the incidence of bacterial pathogens on fresh produce. Due to multiple factors that can cause cross-contamination of fresh produce during harvesting and processing such as contaminated harvesting equipment, knives, workers’ hands or gloves, containers such as bins, boxes, buckets, washing and sanitizing, packaging, storing and so on, we recommend best practices at the postharvest stage so as to improve their microbial quality and safety. Some of these include postharvest washing, irradiation of the fresh produce, ozone, chlorine treatment and high-pressure processing. We also recommend that more research on the source tracking of the pathogens occurring in irrigation water, agricultural soil and fresh produce be carried out to ascertain the origin of these pathogens and possible elimination strategies.

## Figures and Tables

**Table 1:** Some fresh produce associated disease outbreaks and their bacterial etiological agents between 2005 and 2019.

Fresh Produce	Bacterial Pathogens	Number of Cases (Mortalities)	Country	Year	References
Fresh papayas	*Salmonella* Uganda	71 (0)	USA	2019	[36]
Pre-cut melon	*Salmonella* Carrau	117 (0)	USA	2019	[37]
Romaine lettuce	STEC *E. coli* O157:H7	62 (0)	USA	2018	[38]
Leafy greens	*E. coli* O157:H7	25 (1)	USA	2018	[39]
Pre-cut melons	*Salmonella adelaide*	77 (0)	USA	2018	[40]
Alfalfa and raw clover sprouts	*E. coli*	59 (0)	USA	2017	[24]
Spinach	*E. coli*	199 (3)	USA	2017	[24]
Apples	*Listeria monocytogenes*	35 (7)	USA	2017	[24]
Bean sprouts	*Listeria monocytogenes*	5 (2)	USA	2017	[24]
Cantaloupe	*Listeria monocytogenes*	147 (33)	USA	2017	[24]
Alfalfa and raw clover sprouts	*Salmonella* spp.	506 (0)	USA	2017	[24]
Cucumbers	*Salmonella* spp.	991 (6)	USA	2017	[24]
Bean sprouts	*Salmonella* spp.	115 (0)	USA	2017	[24]
Mangoes	*Salmonella* spp.	127 (0)	USA	2017	[24]
Papayas	*Salmonella* spp.	106 (0)	USA	2017	[24]
Jalepenos and serrano peppers	*Salmonella* spp.	1442 (2)	USA	2017	[24]
Cantaloupe	*Salmonella* spp.	332 (3)	USA	2017	[24]
Tomatoes	*Salmonella* spp.	111 (0)	USA	2017	[24]
Lettuce	*E. coli*	34 (NS)	Canada	2017	[24]
Cantaloupe	*Salmonella* spp.	NS	Canada	2017	[24]
Watercress	*E. coli* O157	NS	UK	2016	[41]
Lettuce, cucumber	*E. coli* 096	50 (NS)	UK	2016	[41]
Alfalfa sprouts	STEC *E. coli* O157:H7	11 (0)	USA	2016	[42]
Frozen vegetables	*Listeria monocytogenes*	9 (3)	USA	2016	[43]
Alfalfa sprouts	*Salmonella* Muenchen (25 people) and *Salmonella* Kentucky (1 person)	26 (0)	USA	2016	[44]
Packaged salads	*Listeria monocytogenes*	19 (1)	USA	2016	[45]
Imported cucumbers	*Salmonella* Poona	907 (6)	USA	2015	[46]
Caramel apples	*Listeria monocytogenes*	35 (7)	USA	2014	[47]
Beans sprouts	*Salmonella* Enteritidis	115 (0)	USA	2014	[48]
Raw clover sprouts	STEC *E. coli* O121	19 (0)	USA	2014	[49]
Lettuce, cucumber	Enteroinvasive *E. coli* O96	50	UK	2014	[50]
Salads	*Salmonella* Singapore	4	UK	2014	[50]
Watercress	*Verocytotoxin-producing E. coli* O157	NS	UK	2013	[50]
RTE salads	STEC *E. coli* O157:H7	33 (0)	USA	2013	[51]
Imported cucumbers	*Salmonella* Saintpaul	84 (0)	USA	2013	[52]
Organic Spinach and Spring Mix Blend	STEC *E. coli* O157:H7	33 (0)	USA	2012	[53]
Mangoes	*Salmonella* Braenderup	127 (0)	USA	2012	[54]
Cantaloupe	*Salmonella* Typhimurium and *Salmonella* Newport	261 (3)	USA	2012	[55]
Raw clover sprouts	STEC *E. coli* O26	29 (0)	USA	2012	[56]
Romain lettuce	*E. coli* O157:H7	58	USA	2011	[57]
Cantaloupes	*Listeria monocytogenes*	147 (33)	USA	2011	[58]
Whole, freshly imported papayas	*Salmonella* Agona	106	USA	2011	[59]
Alfalfa sprouts and spicy sprouts	*Salmonella* Enteritidis	25 (0)	USA	2011	[60]
Cantaloupe	*Salmonella* Panama	20 (0)	USA	2011	[58]
Vegetable sprouts	*E. coli* O104:H4	3911 (47)	Europe	2011	[61,62]
Alfalfa sprouts	*Salmonella I 4,[5],12:i:-*	140 (0)	USA	2010	[63]
Raw alfalfa sprouts	*Salmonella* Newport	44 (0)	USA	2010	[64]
Romaine lettuce	*E. coli* O145	26 (0)	USA	2010	[65]
Raw alfalfa sprouts	*Salmonella* Saintpaul	234 (0)	USA	2009	[66]
Raw produce	*Salmonella* Saintpaul	565 (2)	USA	2008	[67]
Peppers	*Salmonella*	1442 (2)	USA, Canada	2008	[68,69]
Cantaloupes	*Salmonella* Litchfield	51 (0)	USA	2008	[70]
Lettuce	*E. coli* O157:H7	134	USA, Canada	2008	[71]
Basil	*Salmonella*	32	UK	2008	[32]
Baby spinach	*Salmonella*	354	Europe	2007	[72]
Basil	*Salmonella*	51	North America, Europe	2007	[73]
Baby carrots	*Shigella sonnei*	230	Australia, Europe	2007	[74]
Alfalfa sprouts	*Salmonella*	45	Europe	2007	[75]
Tomatoes	*Salmonella* Typhimurium	183 (0)	USA	2006	[76]
Fresh spinach	*E. coli* O157:H7	199 (3)	USA	2006	[77]
Alfalfa sprouts	*Salmonella*	125	Australia	2006	[78]
Cantaloupe	*Salmonella*	115	Australia	2006	[79]
Mung bean sprouts	*Salmonella*	592	Canada	2005	[80]
Tomatoes	*Salmonella*	459	USA	2005	[81]

**Table 2:** Types of beta-lactamases that mostly occur in Enterobacteriaceae based on the classification of Ambler and the Bush–Jacoby–Medeiros [267].

Ambler Classification	Bush–Jacoby–Medeiros Classification	Distinctive Substrate	Inhibitor	Representative Enzyme
C	1	cephalosporins	none	*Amp*C
A	2b	penicillins, early cephalosporins	beta-lactamase inhibitors	TEM-1, TEM-2, TEM-13, SHV-1
A	2be	extended-spectrum cephalosporins and aztreonam	beta-lactamase inhibitors	TEM-3, SHV-2, PER, VEB, CTX-M-15
D	2	cloxacillin	beta-lactamase inhibitors	OXA-1, OXA-10
D	2de	extended-spectrum cephalosporins	beta-lactamase inhibitors	OXA-11, OXA-15
D	2df	carbapenems	beta-lactamaseinhibitors	OXA-23, OXA-48
A	2f	carbapenems	beta-lactamase inhibitors	KPC, IMI, SME, NMC
B	3a	carbapenems	EDTA	MBL
